# A Systematic Review of the Management of Knee Osteoarthritis by Proximal Fibular Osteotomy in the Indian Population

**DOI:** 10.7759/cureus.53638

**Published:** 2024-02-05

**Authors:** Aditya Pundkar, Sandeep Shrivastav, Rohan Chandanwale, Ankit M Jaiswal, Saksham Goyal

**Affiliations:** 1 Orthopaedics, Jawaharlal Nehru Medical College, Datta Meghe Institute of Higher Education and Research, Wardha, IND

**Keywords:** safety, effectiveness, management, indian population, proximal fibular osteotomy, knee osteoarthritis

## Abstract

This systematic review aims to assess the management of knee osteoarthritis through proximal fibular osteotomy (PFO) in the Indian population by synthesizing data from various prospective cohort and interventional studies. We seek to provide an overview of the effectiveness and safety of PFO as a treatment modality and offer insights into its potential implications for clinical practice in India. A systematic search strategy was employed, targeting multiple medical databases to identify relevant studies published from 2018 to 2023. Inclusion criteria encompassed studies involving Indian patients with medial compartment knee osteoarthritis and varus deformity who underwent PFO. Data were extracted and evaluated according to the Newcastle-Ottawa Scale for observational studies. Eight studies were included in this review, each displaying varying designs, patient populations, and follow-up duration. The findings consistently indicated that PFO improved pain, knee function, and radiological outcomes, such as knee joint space and tibio-femoral angles. These improvements were generally sustained over several months to a year. The available evidence underscores the potential of PFO as a promising intervention for managing knee osteoarthritis in the Indian population, particularly in patients with medial compartment involvement and varus deformity. While these results are promising, the limitations inherent in the current literature, including study design variations and small sample sizes, necessitate further research with more extensive and diverse patient populations. This systematic review provides valuable insights for healthcare professionals and researchers, highlighting the need for more rigorous investigations and supporting the consideration of PFO as a viable treatment option for knee osteoarthritis in India.

## Introduction and background

Osteoarthritis (OA) of the knee joint is a prevalent and disabling musculoskeletal condition affecting millions globally, posing a substantial burden on the Indian population [1]. The gradual deterioration of articular cartilage, accompanied by pain, joint stiffness, and functional limitations, adversely impacts quality of life and escalates healthcare expenditures [2]. With the aging and evolving lifestyle of the Indian populace, the anticipated increase in knee OA necessitates effective management strategies tailored to the unique characteristics of this population [3].

Proximal fibular osteotomy (PFO), a surgical procedure designed to redistribute mechanical loads in the knee joint, has emerged as a potential therapeutic approach for addressing knee OA [4]. This intervention involves surgically repositioning the fibular head, thereby altering knee loading patterns, with the potential benefits of alleviating pain and enhancing joint function [4]. It is essential to highlight how PFO compares to conventional non-surgical modalities and the limitations it circumvents. Various non-pharmacological techniques, including intra-articular injections, non-steroidal anti-inflammatory drugs (NSAIDs), physical therapy, and weight loss, have demonstrated efficacy as treatment options for knee OA [5]. While these modalities can improve function, reduce pain, and address mobility and stiffness in knee OA, they may fall short of fully restoring the condition. In contrast, PFO offers a surgical approach that specifically aims to redistribute mechanical loads in the knee joint. Unlike some non-surgical modalities that primarily manage symptoms, PFO targets the mechanical aspect of the condition, potentially providing more sustainable relief by addressing the root cause of OA. This surgical intervention may offer advantages in cases where non-surgical modalities have limitations, especially in patients with medial compartment involvement and varus deformity [4,5]. The limitations of non-surgical modalities, such as potential incomplete restoration of the knee condition and the need for ongoing management, underscore the importance of exploring alternative interventions like PFO. While non-surgical options remain valuable, the surgical repositioning of the fibular head in PFO offers a unique approach that may provide more profound and lasting benefits for specific subsets of patients [4,5].

This systematic review aims to comprehensively evaluate the existing evidence concerning the management of knee OA through PFO in the Indian population. The goal of synthesizing relevant study data is to present a consolidated overview of the literature, assess evidence quality, and identify gaps in understanding the effectiveness and safety of this treatment modality. Timely and pertinent, this review addresses a critical healthcare concern in India, contributing to the global discourse on knee OA management. Its potential impact extends to informing clinical practices, guiding future research endeavors, and ultimately enhancing the quality of life for those grappling with knee OA in the Indian context.

## Review

Methods

**Figure 1:**
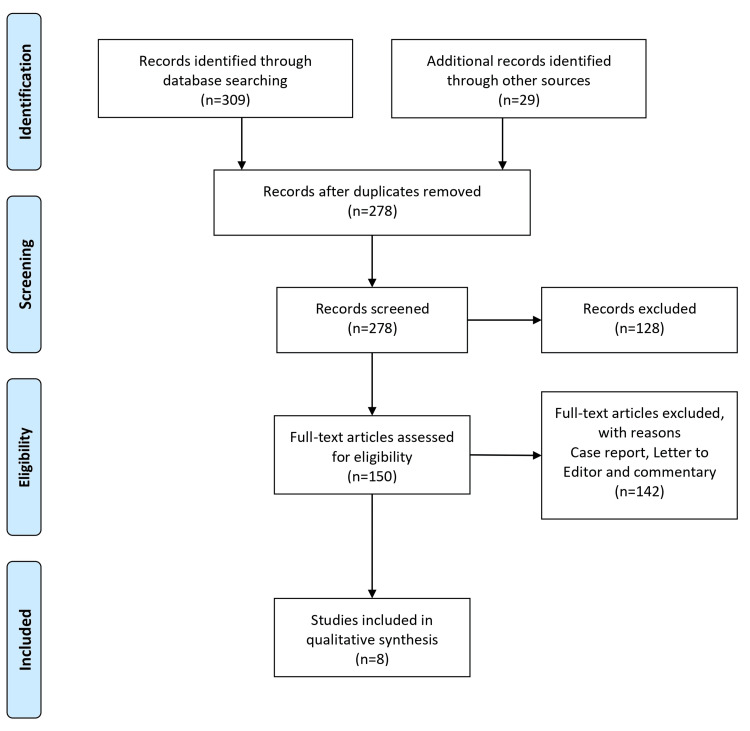
Representation of selection of articles for systematic review

In conducting this systematic review, a meticulous and structured approach was employed to ensure transparency, rigor, and reliability throughout the review process. The review strictly adhered to established guidelines and standards, specifically following the Preferred Reporting Items for Systematic Reviews and Meta-Analyses (PRISMA) [6]. The process commenced with developing a systematic search strategy with a medical librarian. Multiple databases, including PubMed, MEDLINE, Embase, and Cochrane, were systematically searched for articles published from 2018 to June 2023. The search terms utilized a combination of Medical Subject Headings (MeSH) and keywords related to knee osteoarthritis, proximal fibular osteotomy, and the Indian population (Figure 1).

Predefined inclusion and exclusion criteria were rigorously applied to ensure the inclusion of relevant studies. Inclusion criteria encompassed original research articles conducted on Indian populations, examining the use of PFO as a treatment for knee OA, and published in English. Conversely, case reports, reviews, and non-human studies were explicitly excluded. The identified articles underwent a thorough two-stage screening process. Two independent reviewers initially screened titles and abstracts to assess relevance. Potentially relevant articles then underwent a full-text review, with final eligibility determined through consensus in case of disagreements. A standardized form was utilized for data extraction to collect pertinent information from the included studies systematically. This included study characteristics, participant demographics, intervention specifics, outcome measures, and results. Additionally, each study's quality and risk of bias were assessed using appropriate tools, such as the Newcastle-Ottawa Scale for observational studies [7], with two reviewers independently evaluating study quality (Table 1).

**Table 1:** Summarizing the risk of bias assessment for each of the included studies using the Newcastle-Ottawa Scale (NOS)

Study Title and Reference	Selection	Comparability	Outcome	Risk of Bias
Kumar et al., 2021 [8]	★★★★	★★	★★★★	Low
Sabir et al., 2021 [9]	★★★★	★★	★★★	Low
Huda et al., 2020 [10]	★★★★	★★	★★★★	Low
Laik et al., 2020 [11]	★★★★	★★	★★★★	Low
Rai et al., 2019 [12]	★★★★	★★	★★★	Low
Gopi et al., 2019 [13]	★★★★	★★	★★	Low
Gandhi et al., 2022 [14]	★★★★	★★	★★★	Low
Nandi et al., 2021 [15]	★★★★	★★	★★★	Low

Results

Table 2 summarizes eight selected studies conducted in various regions of India, focusing on the impact of PFO on knee OA across diverse patient populations. These studies employed different designs and exclusion criteria but predominantly involved patients with medial compartment knee OA and varus deformity, classified as Kellgren and Lawrence grade II and III (K-L Grade). Outcome assessments encompassed measures such as Visual Analog Scale (VAS) scores, Knee Society Scores (KSS), tibio-femoral angles (FTA), and others. Follow-up periods ranged from immediate post-op assessments to two years. In summary, these studies collectively enhance our understanding of PFO as a potential management option for knee OA in the Indian population.

**Table 2:** Characteristics of included studies OA: Osteoarthritis; VAS: Visual Analog Scale; ML Ratio: Medial to lateral knee joint space ratio; KL: Kellgren-Lawrence; AKSS: American Knee Society Score; FTA: Tibio-Femoral Angle; WOMAC: Western Ontario and McMaster universities osteoarthritis Index; KOA: Knee Osteoarthritis; PFO: Proximal Fibular Osteotomy; KSS: Knee Spacing Score; JCA: Joint Conversion Angle; OKS: Oxford Knee Score; NA: Not Available

Sr. No.	Study ID	Study Design	Details of Participants	Exclusion Criteria	Grade of Osteoarthritis	Details of Outcome Scores	Follow-up	Improvements
1	Kumar et al., 2021 [8], Lucknow, India	Prospective cohort study Hospital setting, August 2018 to July 2019	Patient medial joint OA of the knee who underwent PFO N=21 Mean age: 58.85 ±6.94 years	Patients with incomplete records, Patients with concomitant arthritis due to any other cause (rheumatoid, seronegative OA) Post-traumatic arthritis of the knee H/o ligament or meniscus injury of the knee, Patients with clinical valgus deformity of knees	Grade: 1-4	VAS ML ratio KL AKSS	Three months and one year	VAS score AKSS ML ratio was observed to be maintained for a period of one year
2	Sabir et al., 2021 [9], Aligarh, India	Prospective study Tertiary Care Hospital, November 2017 to November 2019	Patients with degenerative medial compartmental osteoarthritis knee, varus deformity < 15 and Kellgren and Lawrence grade II and grade III (K-L Grade) N=32 Mean age 48.4 years	Bi- or tri-compartmental osteoarthritis, Flexion deformity Genu valgum Inflammatory arthritis Post-traumatic arthritis Previous knee surgery, Body mass index > 30.	KL Grades II and III	VAS KSS Lateral FTA Joint Space ratio	1, 3, 6, 12 months and then annually	VAS Score KSS Clinical and Functional
3	Huda et al., 2020 [10], UP, India	Prospective interventional study Hospital setting, January 2017 to December 2017	Patients with medial compartment osteoarthritis of knee falling in Grade II and Grade III of Kellgren–Lawrence classification N=42 Age: 40 years and older	Not willing to enroll in the study Post-traumatic knee osteoarthritis Radiological evidence of OA in the lateral or patellofemoral compartment Inflammatory joint disease Previous operations around the knee, flexion deformity more than 15° Varus deformity > 15° BMI > 25	KL Grades II and III	VAS WOMAC Lateral FTA	3, 6, and 12 months.	VAS scores WOMAC scores
4	Laik et al., 2020 [11], Jamshedpur, India	Prospective interventional, cohort study Hospital setting, August 2017 to August 2019	Patients with knee pain and difficulty in walking due to medial compartment osteoarthritis N=30 Mean age: 55.80±4.41 yrs	Genu valgus Lateral compartment OA knee Bone to bone contact on weight-bearing X-ray Acute major trauma Inflammatory joint disease Malignant tumors Patient not fit for surgery (abnormal liver or renal functions) Patient not willing for surgery	NA	VAS Tegner Lysholm Knee score X rays FTA	Immediate post-op, at 14th postop at6 weeks, at three months, six months, and two years	Tegner Lysholm knee score
5	Rai et al., 2019 [12], Varanasi, India	Short-term study Hospital setting, June 2016 to April 2018	Patients with moderate to severe symptomatic medial compartment OA of the knee N=38 Mean age: 52.26 yrs	Post-traumatic knee OA, Inflammatory joint disease, Valgus deformity of knee	NA	VAS AKSS Knee Joint Space FTA JCA	Three months	FTA Medial Joint Space
6	Gopi et al., 2019 [13], Chennai, India	Short-term prospective cohort study GMC, June 1, 2018, and June 30, 2019	Bilateral or unilateral bicompartmental OA of the knee, with or without varus deformity N=18	Tricompartmental OA of the knee Inflammatory arthritis Posttraumatic arthritis Previous septic arthritis and knee surgery	NA	VAS Lysholm knee score	2^nd^ day and six months	Lysholm knee score
7	Gandhi et al., 2022 [14], Nagpur, India	Prospective interventional study GMC, November 2017 to October 2020	Patient with Knee pain with medial joint line pain having visual analogue score 5-9, BMI less than 30 kg/m^2^, with Grades 1, 2 and 3 of knee OA K-L and genu varus up to 15º N=58 Age between 45-60 yrs	Patients with genu valgum Inflammatory arthritis Acute trauma Tumours Patello-femoral arthritis	K-L Grades 1 & 2	VAS KSS FTA	1, 3, 6, and 12 months	KSS
8	Nandi et al., 2021 [15], India	Prospective study Hospital setting, December 2017 to December 2020	Patients with medial unicompartmental osteoarthritis of the knee treated with PFO N=50 Mean age: 48.20 yrs	NA	KL Stage 3	OKS	1, 2, and 6 months	PFO has been associated with good outcomes and fewer complications

Overall, we included eight studies [8-15], all prospective cohort studies and interventional studies carried out at different locations in India. In each of these studies, investigations evaluated the existing evidence concerning the management of knee OA through PFO in the Indian population. Across the included studies [8-15], 289 participants were included and recruited patients with medial compartment OA of the knee. All the included studies [8-15] detailed the management of knee OA through PFO. Among the included studies, seven studies [8-14] assessed VAS score, three studies [8,9,12] assessed American Knee Society Score (AKSS), six studies assessed [8-12,14] FTA, one study [14] assessed KSS, and another one study [15] assessed Oxford Knee Scores (OKS).

Discussions

OA of the knee is a significant global health concern, particularly impacting the Indian population [16]. With a growing aging demographic and lifestyle changes, effective management strategies for knee OA are imperative. PFO has emerged as a potential intervention for knee OA, aiming to redistribute mechanical loads in the knee joint [17]. This systematic review seeks to synthesize existing evidence on the effectiveness and safety of PFO in Indian populations with knee OA.

Ashraf et al. [18] conducted a systematic review focusing on clinical studies in Asian countries, exploring outcomes associated with PFO as a potential treatment for knee OA, especially in the medial joint. Their comprehensive analysis led to the promising conclusion that PFO statistically significantly be a viable option for managing knee OA in specific patients as results revealed significant improvement in the VAS and the AKSS functional score and also the femoro-tibial and hip knee ankle angles also demonstrated positive changes.

The studies included in this systematic review exhibited variations in patient characteristics, study design, and geographic locations across different regions in India. These variations are crucial when interpreting findings and assessing the generalizability of results [8-15]. The systematic review found that studies consistently reported various clinical outcome measures, including VAS scores, KSS, OKS, Western Ontario and McMaster Universities Osteoarthritis Index (WOMAC), and more. These measures allowed for assessing pain relief, functional improvement, and quality of life after PFO [8-15].

Structural outcomes, encompassing radiological assessments and anatomical changes, were a key focus in the studies included in this systematic review. Various measurements, including the Medial Lateral Knee Joint Space Ratio (ML ratio), FTA, Joint Conversion Angle (JCA), and Knee Joint Space (KJS), were evaluated across the selected studies [8-15]. The follow-up durations in these studies varied, ranging from a few months to several years. Notably, the findings consistently demonstrated sustained improvements in pain relief and functional outcomes over these extended follow-up periods, suggesting the potential for long-term benefits associated with PFO in managing knee OA [8-15]. However, to address the pertinent question of durability, it is imperative to delve deeper into the specific time frames and the maximum duration of follow-up reported in the individual studies. Understanding whether the observed positive outcomes persist over an extended period is crucial in evaluating the lasting impact of PFO on knee OA. Additionally, exploring whether symptoms may recur over time and investigating the incidence of total knee arthroplasty (TKA) at various follow-up time points will contribute to a more comprehensive understanding of the long-term efficacy and implications of PFO in the Indian population. Overall, the findings of these studies represent a comprehensive picture of the management techniques employed, particularly focusing on PFO within the Indian population. The outcomes suggest that this surgical intervention holds promise in redistributing mechanical loads, potentially relieving pain and improving joint function.

However, in generalizing these results, it is crucial to consider the specific demographic and clinical characteristics of the Indian population under study. Factors such as genetic predispositions, lifestyle, and access to healthcare may influence the outcomes. While the findings offer valuable insights for the Indian context, caution should be exercised when extrapolating these results to a broader, global population.

Sugianto et al. [19] conducted a systematic review and meta-analysis focusing on managing medial compartment knee OA. The results showed significant improvement in knee function and alleviated pain. Complication rates were minimal, with peroneal nerve paresthesia being the most common adverse event. PFO was associated with improved medial/lateral joint space ratio, indicating positive radiological outcomes. The overall conclusion proposes that PFO exhibits potential in managing medial compartment KOA, showcasing significant improvements in both clinical and radiological aspects.

This systematic review acknowledges that the included studies have limitations, such as small sample sizes and the absence of control groups. These studies highlight its potential in redistributing mechanical loads, potentially alleviating pain, and improving joint function. However, the specifics of the Indian population, including genetic factors, lifestyle, and healthcare access, are crucial considerations. At the same time, these results offer valuable insights into the Indian context. Furthermore, additional research, including prospective, multi-center, double-blind, non-randomized, and randomized controlled trials (RCTs) with sufficient power, sample size, and extended follow-up is needed to explore the long-term outcomes and potential complications associated with PFO in the Indian population. To enhance the analytical approach, there is a planned effort to identify patterns, commonalities, and variations in the effectiveness of proximal fibrotomy as a treatment for knee joint OA.

Limitations

This systematic review on PFO for knee OA in the Indian population has limitations, including a few studies, methodological heterogeneity, and a focus solely on the Indian perspective. The absence of RCTs raises concerns about potential bias, and the lack of a comprehensive comparative analysis with other treatment modalities limits practical applicability. These constraints highlight the need for caution in interpreting the findings and emphasize areas for future research to enhance the reliability and global relevance of the evidence.

## Conclusions

In conclusion, this systematic review has significantly synthesized the available evidence on managing knee OA through PFO within the Indian population. Drawing from diverse perspective cohorts and interventional studies conducted across various regions of India, the review sheds light on the potential benefits of PFO for individuals with medial compartment knee OA and varus deformity. While consistently demonstrating the ability of PFO to alleviate pain, enhance knee function, and improve radiological outcomes over several months to a year of follow-up, it is essential to acknowledge the global nature of knee OA as a prevalent health issue. The comment received from reviewers underscores the importance of considering results from global studies to ensure statistical relevance, guide future research, and inform international guidelines. Moreover, the reviewers rightly point out the necessity to clarify how the systematic review contributes to policymaking, distinguishes the advantages of PFO over other modalities, and explores its cost-effectiveness compared to existing options. Future review iterations should address these considerations, providing a more comprehensive and globally applicable perspective that aligns with the broader implications of knee OA management.
